# Investigation of *Staphylococcus aureus* Biofilm-Associated Toxin as a Potential Squamous Cell Carcinoma Therapeutic

**DOI:** 10.3390/microorganisms12020293

**Published:** 2024-01-30

**Authors:** Zi Xin Ong, Bavani Kannan, Anthony R. J. Phillips, David L. Becker

**Affiliations:** 1 Lee Kong Chian School of Medicine, Nanyang Technological University, Singapore 308232, Singapore; 2 Skin Research Institute Singapore, Singapore 308232, Singapore; 3 Nanyang Institute of Technology in Health and Medicine, Interdisciplinary Graduate Programme, Nanyang Technological University, Singapore 639798, Singapore; 4 School of Biological Sciences, Auckland University, Auckland 1030, New Zealand; a.phillips@auckland.ac.nz; 5 National Skin Centre, Singapore 308205, Singapore

**Keywords:** squamous cell carcinoma, *Staphylococcus aureus*, biofilm-conditioned media, transposon mutant library, selective toxin, hemolysin

## Abstract

Cancer therapies developed using bacteria and their components have been around since the 19th century. Compared to traditional cancer treatments, the use of bacteria-derived compounds as cancer therapeutics could offer a higher degree of specificity, with minimal off-target effects. Here, we explored the use of soluble bacteria-derived toxins as a potential squamous cell carcinoma (SCC) therapeutic. We optimized a protocol to generate *Staphylococcus aureus* biofilm-conditioned media (BCM), where soluble bacterial products enriched in the development of biofilms were isolated from a bacterial culture and applied to SCC cell lines. Bioactive components of *S. aureus* ATCC 29213 (SA29213) BCM display selective toxicity towards cancerous human skin SCC-12 at low doses, while non-cancerous human keratinocyte HaCaT and fibroblast BJ-5ta are minimally affected. SA29213 BCM treatment causes DNA damage to SCC-12 and initiates Caspase 3-dependent-regulated cell death. The use of the novel SA29213 *bursa aurealis* transposon mutant library led to the identification of *S. aureus* alpha hemolysin as the main bioactive compound responsible for the observed SCC-12-specific toxicity. The antibody neutralisation of Hla eradicates the cytotoxicity of SA29213 BCM towards SCC-12. Hla displays high SCC-12-specific toxicity, which is exerted primarily through Hla-ADAM10 interaction, Hla oligomerisation, and pore formation. The high target specificity and potential to cause cell death in a controlled manner highlight SA29213 Hla as a good candidate as an alternative SCC therapeutic.

## 1. Introduction

Squamous cell carcinoma (SCC) is the cancer of squamous epidermal keratinocytes and adnexal structures and usually develops on sun-exposed skin, such as the head and neck. Cutaneous SCC accounts for approximately 20% of all skin cancers [1] and reportedly affects approximately 2.2 million people at any one time [2]. Although SCC is second to basal cell carcinoma (BCC) in prevalence amongst non-melanoma skin cancers (NMSCs) [3,4], it is more likely to metastasize and cause mortality [5]. One of the primary causes of DNA damage in SCC is ultraviolet B (UVB) radiation [6,7]. Persons of light skin tone, such as Fitzpatrick skin types I and II, have a high propensity to develop SCC [8]. Furthermore, immunocompromised individuals, such as organ transplant recipients [9,10] and HIV/AIDS patients [11], have grossly increased chances of developing SCC. Other risk factors include scars, chronic wounds, arsenic exposure, radiation therapy, and previous BCC and HPV infections [8].

Surgical options, such as surgical excision (SE) and Mohs micrographic surgery (MMS), remain the main treatment strategies in high-risk SCCs. The complete removal of the tumour offers high cure rates of up to 95% in SE [12] and 97% in MMS [13,14] for patients with primary SCC. However, these invasive treatment strategies are not ideal for SCCs occurring in regions with low skin laxity. Non-invasive therapeutics, such as imiquimod cream, are viable options if skin abnormalities are detected early [15]. However, these options are often ineffective in treating fully developed SCC [16]. A non- or minimally invasive treatment strategy would be an ideal alternative to surgical options for patients with fully developed SCC in hard-to-excise regions.

Bacteria-derived compounds could offer an alternative solution to traditional cancer treatment strategies, such as radiation or chemotherapy, while potentially allowing for a higher degree of target specificity. Unlike the use of live or attenuated bacteria, the use of isolated and purified bacterial products eliminates the risk of exaggerated infections. The therapeutic potential of bacterial toxins in cancer has been well studied. Exudates from species such as *Pseudomonas* [17] and *Clostridium sporogenes* [18] have been reported to negatively impact cancer cell survivability and proliferation. Several bacterial products, such as botulinum toxin and adenylate cyclase toxin, have been reported to enhance the effects of radiotherapy and chemotherapy on cancer tissues [19,20]. Similarly, various toxins, such as *Pseudomonas* Exotoxin A and Diphtheria toxin, when conjugated to tissue-specific peptides, have been shown to enhance host immune stimulation or toxin tissue specificity [17,21,22]. Recently, skin commensal *Staphylococcus epidermis* was shown to produce 6-*N*-hydroxyaminopurine (6-HAP) that inhibits DNA-polymerase activity, suppressing the growth of B16F10 melanoma [23]. These highlight the largely untapped potential of bacterial toxins as cancer therapeutic agents.

*Staphylococcus aureus* is a Gram-positive pathogenic bacterium that can cause diseases of varying severity, ranging from food poisoning to life-threatening diseases such as sepsis [24]. On the skin, *S. aureus* commonly exists as a commensal bacterium. However, its highly opportunistic nature makes the bacterium a prominent player in various skin diseases, such as impetigo, folliculitis, and, more severely, staphylococcus-scalded skin syndrome (SSSS). In this study, the use of *S. aureus* biofilm-associated soluble products in SCC therapy was explored. Biofilm-conditioned media (BCM) were generated using an optimized protocol, which allowed for high biofilm accumulation and the efficient capture of biofilm-associated secreted products. Our data show that the BCM of *S. aureus* chronic wound isolate SA29213 were highly toxic towards the human SCC cell line SCC-12. Non-cancerous human keratinocyte HaCaT and fibroblast BJ-5ta were, in comparison, less sensitive to SA29213 BCM. Staphylococcal alpha hemolysin (Hla) was found to be the primary source of SA29213 BCM’s toxicity towards SCC-12, triggering a target-specific caspase-dependent cell death in SCC-12 cells. Hla is a beta-barrel pore-forming toxin (PFT) that is responsible for various *S. aureus*-associated malignancies [25], including dermonecrosis [26,27], pneumonia [28,29,30,31,32,33], cornea keratitis [34,35], and sepsis [36,37]. Thorough investigation is needed to further characterize the target specificity of Hla and, subsequently, its suitability as a potential skin cancer therapeutic.

## 2. Materials and Methods

### 2.1. Cell Culture

Human skin SCC-12 (RRID: CVCL_4026) [38], the spontaneously immortalised human keratinocytes HaCaT (RRID: CVCL_0038) [39], and h-TERT-immortalised human fibroblast BJ-5ta (RRID: CVCL_6573) were used. Cell lines were maintained in SCC-12 cell culture media, which were comprised of 1:1 Gibco^TM^ Dulbecco’s Modified Eagle Medium, high glucose, sodium pyruvate (DMEM) to Gibco^TM^ Ham’s F12 Nutrient Mixture (F12) (Thermo Fisher Scientific, Waltham, MA, USA), supplemented with 10% fetal bovine serum (FBS) (HyClone Laboratories, South Logan, UT, USA) and 1% Gibco^TM^ penicillin/streptomycin (P/S) (Thermo Fisher Scientific, Waltham, MA, USA). All cells were grown as monolayers in a 5% CO_2_ humidified chamber at 37 °C.

### 2.2. Bacterial Strains

Wound isolate *Staphylococcus aureus* subsp. *Aureus* Rosenbach (ATCC^®^ 29213^TM^) was purchased from American Type Culture Collection (ATCC, Manassas, VA, USA). Prior to conditioned media generation, an overnight starter culture was prepared (200 rpm, 16 h, 37 °C) in Tryptic soy broth (TSB) (Sigma-Aldrich, Burlington, MA, USA).

### 2.3. Biofilm-Conditioned Media Preparation

**Figure 1:**
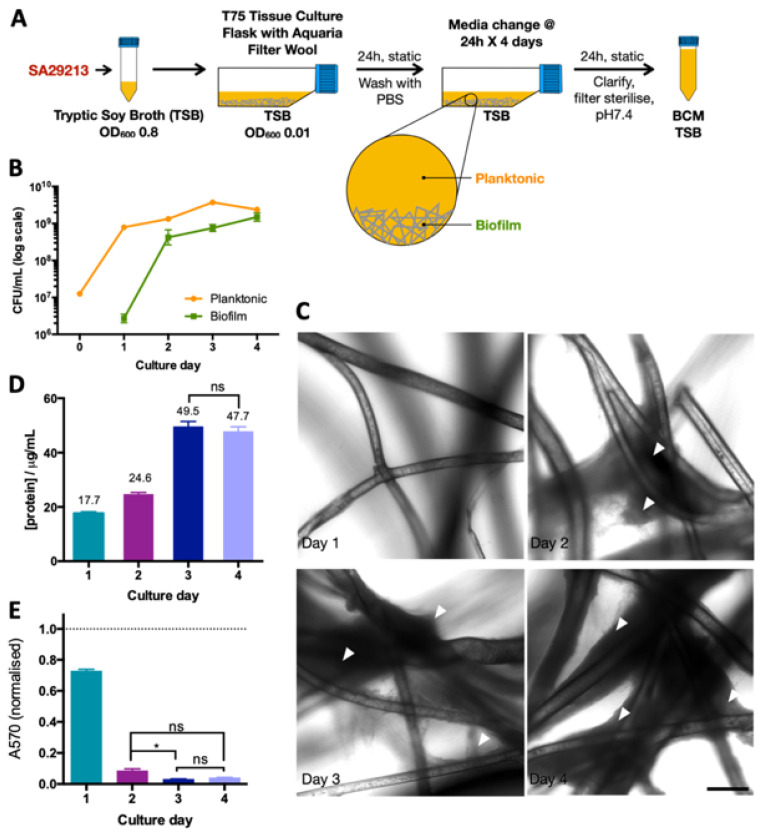
Generation of TSB-based static culture SA29213 BCM. (**A**) Schematic of static BCM generation protocol. BCM was harvested every 24 h for 4 days. Bacteria in suspension was defined as the planktonic population and bacteria adherent on the aquaria filter wool was defined as the biofilm population. (**B**) CFU/mL of SA29213 planktonic and biofilm population of each BCM culture day. CFU was adjusted to per mL of BCM (40 mL). (**C**) SA29213 biofilm accumulation on aquaria filter wool. Observable biofilm accumulation on aquaria filter wool is indicated with white arrows. Image acquired using SPE confocal microscope at 20× magnification. Scale bar represents 1 mm. (**D**) Protein concentration of BCM of each culture day as quantified by Bradford Protein Assay. (**E**) Effect of BCM of each culture day on SCC-12 metabolic activity. Cells were treated with 10% BCM of each culture day. Metabolic activity was assessed at 24 h post treatment using MTT assay. Data are shown as mean ± SEM of three technical replicates. *n* = 1. Cell viability was normalised to control. Two-way ANOVA analyses with post hoc Bonferroni’s multiple comparisons test were performed between the various BCMs and control. (*: *p* ≤ 0.05, ns: *p* > 0.05).

Static Culture Biofilm Conditioned Media (Figure 1A): SA29213 was inoculated at OD_600_ 0.01 in T75 Nunc™ EasYFlask™ Cell Culture Flasks (Thermo Fisher Scientific, Waltham, MA, USA), with a thin layer of aquaria filter wool (0.20 g, 75 cm^2^) containing 40 mL TSB. Cultures were incubated statically at 37 °C, with spent media harvested and replaced with fresh TSB every 24 h for up to 96 h. Spent TSB was clarified via centrifugation (4000 rpm, 40 min, 4 °C), filter-sterilized (0.2 μm pore size), and pH-adjusted to ~7.4. The resultant conditioned media (hereon referred to as BCM) were stored at −80 °C until use. Colony-forming units (CFUs) of biofilm and planktonic population per volume of BCM were enumerated daily via CFU counting. Adherent bacteria on aquaria filter wool were dissociated using sonication in a chilled Elmasonic S 30 (H) water bath sonicator (Elma Schmidbauer GmbH, Singen, Germany) (37 kHz, 10 min per cycle, 3 cycles, 1 min vortex after each cycle). Biofilm accumulation on aquaria filter wool was visualised using brightfield microscopy on Leica SP8 (Leica, Wetzlar, Germany) at 20× magnification.

High-throughput Static Culture Biofilm-Conditioned Media: SA29213 mutants were inoculated using sterile disposable plastic 96-pin replicators into 150 μL of TSB in 96-well flat bottom TC-treated microplate (Corning, Corning, NY, USA). Cultures were incubated shaking for 5 h (150 rpm, 37 °C) or until average turbidity reached OD_600_ 0.05 and static for 48 h at 37 °C in a highly humidified environment without media change. Spent media were filter-sterilised using MultiScreen GV Filter plates (0.22 μm pore size, Merck, Rahway, NJ, USA) and collected onto fresh 96-well microplate. The resultant conditioned media (hereon referred to as BCM) were stored at −80 °C until use. 

Six-well Biofilm-Conditioned Media: SA29213 was inoculated at OD_600_ 0.01 in Nunc™ Cell-Culture-Treated 6-well plates (Thermo Fisher Scientific, Waltham, MA, USA) with a thin layer of aquaria filter wool (0.02 g, 9.5 cm^2^) containing 5 mL TSB. Cultures were incubated statically at 37 °C, with spent media harvested and replaced with fresh TSB every 24 h for 72 h. Spent TSB was clarified via centrifugation (4000 rpm, 40 min, 4 °C) and filter-sterilized (0.2 μm pore size). The resultant conditioned media (hereon referred to as BCM) were stored at −80 °C until use.

Sample Concentration: SA29213 BCM was concentrated via centrifugal ultrafiltration (4 °C, 3000× *g*, 7 min cycles) using Vivaspin^®^ 20 ultracentrifuge unit (Cytiva, Chicago, IL, USA) with 10,000 molecular weight cut-off (MWCO) membrane (Vivaspin 20). BCM was concentrated to the dead-stop volume and washed with 1 sample volume of cold sterile PBS, reconstituted to 2 mL with cold sterile PBS, filter-sterilised, and snap-frozen with liquid nitrogen. Protein concentration of the concentrated BCM was quantified using Quick Start^TM^ Bradford Protein Assay (Bio-Rad, Hercules, CA, USA).

### 2.4. BCM Inactivation

Heat Treatment: Unconcentrated day 3 (D3) BCM was heated at 55 °C for 1 h using a water bath. Heat-treated BCM was cooled to room temperature and applied at 10% onto an SCC-12 monolayer.

*Proteinase K* (*ProK*) *Treatment*: D3 BCM was concentrated and buffer-exchanged using Vivaspin 20. Concentrated BCM was adjusted to protein concentration of 200 µg/mL and digested with 20 µg/mL ProK (Qiagen, Hilden, Germany) overnight at 4 °C. ProK-digested BCM was applied at 10% (protein concentration of 20 µg/mL) along with 0.1 mM PMSF onto SCC-12 monolayer.

### 2.5. BCM Fractionation by Fast Protein Liquid Chromatography (FLPC)

BCM was purified by acetone precipitation. Briefly, 4 volumes of cold acetone (−20 °C) were added to 1 volume of BCM and incubated at −20 °C for 1 h. Protein precipitate was pelleted down (4000× *g*, 30 min, 4 °C), and the acetone was allowed to dry at room temperature for up to 30 min. The protein pellet was resuspended in cold sterile PBS and allowed to dissolve overnight with gentle rolling at 4 °C. Solution was clarified (4000× *g*, 30 min, 4 °C) and concentrated using Vivaspin 20. Sample was buffer-exchanged to PBS (pH 7.4) or IEX-Q Start Buffer (20 mM Tris, 50 mM NaCl, pH 8.5).

Anion-exchange chromatography: Purified and concentrated sample was loaded on a HiTrap Q-XL 5 mL (Cytiva, Marlborough, MA, USA) attached to ÄKTA Pure purification system (Cytiva, Marlborough, MA, USA), which was previously equilibrated with 10 column volumes (CV) of IEX-Q Start Buffer. The column was washed with 10 CV of IEX-Q Start Buffer. BCM was fractionated by step-elution with increasing NaCl concentrations (100, 200, 300, 400, 500 mM, and 1 M) at 5 CV intervals. Fractions containing the peaks of interest were pooled, concentrated using Vivaspin 20, and buffer-exchanged to PBS.

### 2.6. SCC-12/HaCaT Co-Culture

HaCaT cells were stained with Vybrant DiO cell-labelling solution (Life Technologies, Waltham, MA, USA) at 1:200, as per manufacturer’s instructions. Stained HaCaTs were then seeded at 160,000 cells/mL together with 220,000 cells/mL SCC-12 into a 24-well plate 24 h prior to start of experiment.

### 2.7. Live/Dead Fluorescence Microscopy

Time-Lapse Microscopy: Cells were grown overnight on a 24-well plate (200,000 cells/mL) to 90% confluence. Cells were stained with 1 µg/mL Propidium iodide (PI) (Life Technologies, Waltham, MA, USA), 0.5 µg/mL Hoechst 33342 (Life Technologies, Waltham, MA, USA), and 0.5 µg/mL Hoechst 33258 (Life Technologies, Waltham, MA, USA) prior to treatment and imaging. Unless otherwise specified, cells were treated with 2.5 µg/mL BCM. Cell death inhibitors Fer-1 (SML0583, Sigma Aldrich, Burlington, MA, USA), Nec-1 (N9037, Sigma Aldrich, Burlington, MA, USA), and Z-VAD-FMK (G7231, Promega, Madison, WI, USA), and Hla-neutralising anti-staphylococcal α-toxin rabbit whole anti-serum (S7531, Sigma Aldrich, Burlington, MA, USA) were introduced along with BCM treatment. Time-lapse imaging of treated cells was performed for 18 h with Integrated Modulation Contrast (IMC) and fluorescence microscopy at 20× magnification on a Leica DMI6000B inverted microscope (Leica, Wetzlar, Germany) with an attached humidified chamber (37 °C, 5% CO_2_). Images of random duplicate fields per well were captured with Leica DFC3000G every hour. Hoechst fluorescence of nuclei was visualised with A4 filter cube (excitation: 340–380 nm, emission: 450–490 nm). PI fluorescence of dead cells was visualised with Rho filter cube (excitation: 541–551 nm, emission: 565–605 nm). Percentage of live cells was calculated with the following formula.

$$Percentage\;\left(\%\right)\;live\;cell=1-\frac{Dead\;cells\;(PI\;positive)}{Total\;cells\;(Hoechst\;positive)}$$

### 2.8. Cell Viability and Caspase Activity Assay

Cells were grown overnight on 96-well plate (200,000 cells/mL) to 90% confluence. MTT assay was conducted using Cell Proliferation Kit 1 (MTT) (Roche, Basel, Switzerland), and absorbance was measured after incubation with solubilisation buffer for 24 h at OD_570_. Caspase 3/7 activity was assessed using Caspase-Glo 3/7 Assay (Promega, Madison, WI, USA), as per manufacturer’s instructions, and luminescence was read after 2 h incubation with substrate. Cytation™ 3 (BioTek, Singapore) was used to measure absorbance and luminescence. 

### 2.9. Immunocytochemistry

SCC-12 monolayer was grown on glass coverslips (14 mm diameter round) in 24-well plates 24 h prior to the start of experiment. After BCM treatment, cells were fixed using 4% paraformaldehyde (PFA) pH 7.4 for 15 min. Coverslips were washed with cold PBS to remove residual PFA. Cells were permeabilised with 0.1% Triton-X in PBS and blocked with 1% Bovine Serum Albumin (BSA) in PBS overnight. HMGB1 and Cleaved Caspase-3 were probed with rabbit anti-HMGB1 antibody (ab79823, Abcam, Cambridge, UK) (1:400) and rabbit Cleaved Caspase-3 (Asp 175) antibody (#9661, Cell Signalling Technology, Danvers, MA, USA) (1:400) for 1 h at room temperature and goat anti-rabbit Alexa 488 (#7074S, Cell Signalling Technology, Danvers, MA, USA) (1:10,000) for 1 h at room temperature. Cells were counterstained with Rhodamine Phalloidin (1:300) for 30 min and DAPI (1:10,000) for 15 min. Coverslips were mounted onto Menzel Gläser polysine glass slides with Citifluor mountant media and sealed with nail varnish. Mounted slides were stored at 4 °C. Images of random triplicate fields per coverslip were captured with Leica SP8 (Leica, Wetzlar, Germany) at 40× magnification.

### 2.10. Western Blot

Mammalian cells were lysed with cold RIPA lysis and extraction buffer (Life technologies, Waltham, MA, USA, Cat No. 89900), complete with 0.2 mM EDTA, 10 µM DTT, and 1 × Protease Inhibitor cocktail (Nacalai Tesque, Kyoto, Japan). Protein concentration was quantified using Pierce BCA Protein Assay Kit (Thermo Fisher Scientific, Waltham, MA, USA). The, 20 µg of cell lysate protein or whole BCM was electrophoresed and transferred to nitrocellulose membrane using iBolt 2 Dry Blotting System (Thermo Fisher Scientific, Waltham, MA, USA) (20 V, 7 min). Western blots were blocked with 5% non-fat milk for 1 h at room temperature. Phospho-Histone H2A.X and Hla were probed with rabbit anti-Phospho-H2A.X (#2577, Cell Signalling Technology, Danvers, MA, USA) (1:2000) and anti-staphylococcal α-toxin rabbit whole anti-serum (S7531, Sigma Aldrich, Burlington, MA, USA) (1:1000), respectively, in 5% non-fat milk overnight at 4 °C. Membranes were washed with PBS with 0.1% Tween-20 and probed with goat anti-rabbit (#7074S, Cell Signalling Technology, Danvers, MA, USA) (1:10,000) secondary antibody conjugated with horseradish peroxidase (HRP) (NA931V, GE Healthcare Life Science, Marlborough, MA, USA) (1:10,000) in 5% non-fat milk for 1 h at room temperature. Membranes were washed, and the secondary antibody was visualized with ECL Ultra (TMA-6) chemiluminescent reagent (Lumigen, Southfield, MI, USA).

### 2.11. Transposon Mutagenesis

Dh5α/pBursa and Dh5α/pFA545 were kind gifts from Professor Missiakas (University of Chicago). SA29213 *bursa aurealis* transposon library was prepared with reference to published protocol by Bae et al., with modifications [40]. Plasmids pFA545 and pBursa were individually passaged through the *E. coli* IM01B strain before transformation sequentially into SA29213 electrocompetent cells. Successfully transformed colonies were selected using tryptic soy agar (TSA; Sigma Aldrich) containing 10 μg/mL chloramphenicol and 2.5 μg/mL tetracycline (TSA_Chl10Tet2.5_) and incubated at 30 °C. Single colonies of SA29213/pFA545/pBursa were diluted 4 times using sterile Milli-Q water pre-heated to 43 °C, spread onto TSA plates containing 10 μg/mL erythromycin (TSA_Erm10_), and incubated at 43 °C for 5–7 days until individual colonies emerge. Single colonies post-mutagenesis were individually streaked onto fresh TSA and incubated at 37 °C overnight or until individual colonies emerged. Single colonies of each mutagenesis sample were inoculated into TSB in 96-well 2 mL deep well plate (Axygen, Union City, CA, USA), sealed with Breathe-easy^®^ sealing membrane (Sigma Aldrich), and incubated overnight at 37 °C in a shaking incubator at 220 rpm. Successful mutagenesis was confirmed using antibiotic patching, where individuals resistant to 10 μg/mL erythromycin and susceptible to 10 μg/mL chloramphenicol and 2.5 μg/mL tetracycline were included in the library collection. Glycerol stocks of each individual of the library were prepared using 25% glycerol and stored at −80 °C.

### 2.12. Reverse-Transcription Quantitative Polymerase Chain Reaction (RT-qPCR)

Biofilm RNA was extracted from bacteria adhering to aquaria filter wool during the generation of static-culture 6-well BCM 3 days post-inoculation. Bacteria were digested with 0.05 mg/mL lysostaphin in TE buffer for 1 h and lysed with Trizol Reagent (Thermo Fisher Scientific, Waltham, MA, USA) according to manufacturer’s recommendations. Reverse transcription and gDNA removal were conducted using ReverTra Ace™ qPCR RT Master Mix with gDNA Remover (Toyobo, Scottsboro, AL, USA). Quantitative PCR was conducted using Luna^®^ Universal qPCR Master Mix (New England Biolabs, Ipswich, MA, USA) using the following primers: Hla-F: 5′-AAG GCC GCC AAT TTT TCC TG-3′; Hla-R: 5′-AGT GGT TTA GCC TGG CCT TC-3′. RT-qPCR data were analysed according to the double-delta Ct analysis method.

### 2.13. Liquid Chromatography Tandem Mass Spectrometry (LC-MS/MS) and Protein Identification (ID)

LC-MS/MS and protein ID were conducted in collaboration with NTU School of Biological Sciences (SBS) Mass Spectrometry facility. Samples were subjected to in-gel tryspin digestion. Peptides were separated and analysed using a Dionex Ultimate 3000 RSLCnano system coupled to a Q Exactive instrument (Thermo Fisher Scientific, Waltham, MA, USA). Separation was performed on a Dionex EASY-Spray 75 μm × 10 cm column packed with PepMap C18 3 μm, 100 Å (Thermo Fisher Scientific) using solvent A (0.1% formic acid) and solvent B (0.1% formic acid in 100% ACN) at flow rate of 300 nL/min with a 60 min gradient. Peptides were then analysed on a Q Exactive apparatus with an EASY nanospray source (Thermo Fisher Scientific, Waltham, MA, USA) at an electrospray potential of 1.5 kV. Raw data files were processed and searched using Proteome Discoverer 2.1 (Thermo Fisher Scientific) against database constructed for SA29213 (NCBI Accession: PRJNA292059). The raw LC-MS/MS data files were loaded into Spectrum Files (default parameters set in Spectrum Selector). The Mascot algorithm was then used for data searching to identify proteins using the following parameters: missed cleavage of two; dynamic modifications were oxidation (+15.995 Da) (M) and phosphorylation (+79.966 Da) (S, T, Y). The static modification was Carbamidomethyl (+57 Da) (C). Percolator was applied to filter out the false MS2 assignments at a strict false-discovery rate of 1% and relaxed false-discovery rate of 5%. 

## 3. Results

### 3.1. S. aureus BCM Displays Selective Toxicity towards Cancerous Keratinocytes

To enrich *S. aureus*-secreted factors upon biofilm accumulation, we designed and optimised a BCM generation protocol that allows for both a high output of conditioned media and a favourable biofilm-forming environment. The biofilm was cultured in a T75 tissue culture flask lined with a thin layer of aquaria filter wool. This allows for biofilm accumulation in a controlled sterile environment, with a large air–liquid interface for sufficient aeration and surface area for bacterial adhesion. The biofilm was cultured for 4 days in tryptic soy broth (TSB), with a media change every 24 h (Figure 1A) to encourage a higher degree of biofilm maturity, without the risk of sloughing resulting from nutrient deprivation. Biofilm growth was documented everyday through colony-forming unit (CFU) enumeration and confocal imaging of aquaria filter wool. Static culturing conditions promoted biofilm accumulation, with large biofilm masses apparent on filter wool after 48 h of culturing (Figure 1B), and both the planktonic and biofilm populations reaching 10^9^ CFU/mL 72 h post-inoculation (Figure 1C). The comparable CFU/mL levels of planktonic culture and biofilm suggest a biofilm-favourable culturing condition.

The accumulation of biofilm from prolonged culturing was associated with the increase in BCM protein concentration and SCC-12 toxicity (Figure 1D). The increase in BCM protein concentration plateaued at 3 days post-inoculation. Similarly, SCC-12-specific toxicity reached a maximum at 3 days post-inoculation. Treatment with day 3 and 4 BCM resulted in the largest reduction in SCC-12 metabolic activity at 10% treatment (Figure 1E). Day 3 and 4 BCM were considered to be representative of secreted products of mature *S. aureus* biofilm and were pooled for use in subsequent experiments.

**Figure 2:**
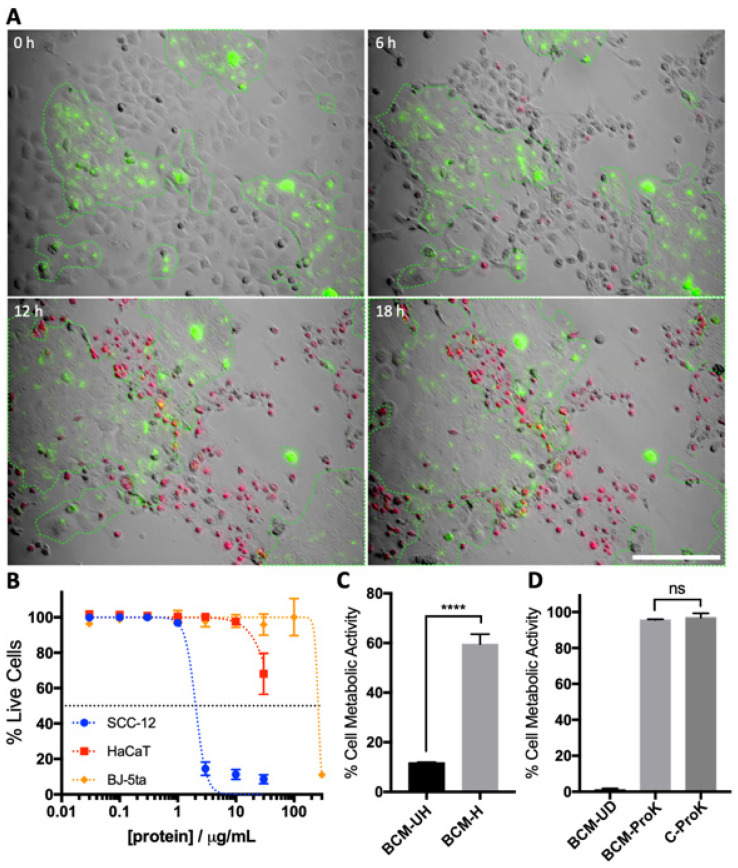
Low-dose BCM, while highly toxic towards SCC-12, caused minimal HaCaT cell death. (**A**) Co-culture of HaCaT (green) and SCC-12 treated with 2.5% BCM. SCC-12 was shown to readily accumulate PI while HaCaT remain viable. HaCaT colonies are highlighted with a green dotted line. Image taken at 0, 6, 12, and 18 h post-treatment at 20× magnification. HaCaT was dyed with Vybrant DiO (green). Dead cells were stained with PI (red). Scale bar represents 150 µm. (**B**) Cell death profile of SCC-12, HaCaT, and BJ-5ta upon BCM treatment was assessed using live/dead fluorescent time-lapse microscopy. Cells were treated with 0.03 to 300 µg/mL concentrated SA29213 BCM. Cell lysis was observed in SCC-12 and HaCaT treated with 100 and 300 µg/mL concentrated SA29213 BCM and was hence excluded from cell death quantification. Dotted line represents non-linear regression. *n* = 3. (**C**) SCC-12 cell viability upon 24 h treatment with heat-treated BCM as assessed by MTT assay. Unconcentrated D3 BCM was heated for 1 h at 55 °C and applied onto SCC-12 at 10% (BCM-H). (**D**) SCC-12 cell viability upon 24 h treatment with undigested BCM (BCM-UD) ProK digested BCM (BCM-ProK), and ProK digested media (C-ProK) as assessed by MTT assay. Data are shown as mean ± SEM of three technical replicates. *n* = 1. Cell viability was normalised to control. Two-way ANOVA analyses with post hoc Bonferroni’s multiple comparisons test were performed between the various BCM treatments and control (****: *p* ≤ 0.0001, ns: *p* > 0.05).

The selective toxicity of SA29213 BCM towards SCC-12 was first demonstrated with a co-culture of SCC-12/HaCaT. The use of a cancerous and non-cancerous keratinocyte co-culture allows for the modelling of the tumour-edge environment. To differentiate the two cell populations, HaCaT cells were stained with Vybrant DiO and seeded alongside unstained SCC-12 cells. Low-dose BCM treatment of SCC-12/ HaCaT co-culture resulted in SCC-12 cell death, while HaCaT cells remained viable (Figure 2A). At 18 h post-treatment of 2.5% BCM, we observed a large proportion of the SCC-12-accumulated PI dye (red), indicating cell death. PI-positive SCC-12 produced giant membrane blebs, unlike apoptosis-associated membrane blebbing, suggesting membrane injury. The lack of PI staining in Vybrant DiO-stained HaCaT (green) suggested that the cells remained viable despite being in close proximity to dead SCC-12. Furthermore, mitosis was observed in HaCaT colonies, despite the presence of BCM, suggesting that the critical SA29213 BCM dosage for SCC-12 minimally affects the cell viability of HaCaT and that BCM-induced cell death does not cause secondary toxicity towards non-cancerous keratinocytes.

The effect of SA29213 BCM on cell viability of cancerous keratinocytes SCC-12, non-cancerous keratinocytes HaCaT, and non-cancerous fibroblasts BJ-5ta was assessed. The 50% cytotoxic concentration (CC50) of each cell line was determined by quantitating the degree of cell death upon treatment with concentrated SA29213 for 24 h. SCC-12 displayed a much higher susceptibility (CC50 = 2.033 µg/mL) to BCM treatments than non-cancerous HaCaT (CC50 = 48.66 µg/mL) and BJ-5ta (CC50 = 259.2 µg/mL) (Figure 2B). The selectivity index (SI) of SA29213 BCM on SCC-12 was 23.72 and 127.5 for HaCaT and BJ-5ta, respectively.

The heat sensitivity of the bioactive compound was investigated by heating unconcentrated BCM at 55 °C for 1 h. Heat-treated BCM possessed significantly reduced toxicity towards SCC-12, whereby treatment at 10% led to an approximately 60% reduction in cell metabolic activity 24 h post-treatment (*p* < 0.0001) (Figure 2C). This suggests that the bioactive compound rendering toxicity towards SCC-12 is heat labile. To investigate if the bioactive compound is proteinaceous, concentrated BCM were digested with broad-spectrum serine protease Proteinase K (ProK) at a low digestion temperature to degrade all proteins. ProK activity was terminated with the use of serine protease inhibitor phenylmethane sulfonyl fluoride (PMSF) prior to application to cells. Treatment with digested BCM led to no decrease in SCC-12 cell viability, indicating a complete removal of toxic bioactive components in BCM (*p* = 0.5659) (Figure 2D). 

### 3.2. Characterisation of SA29213 BCM Toxicity on Cancerous and Non-Cancerous Keratinocytes

**Figure 3:**
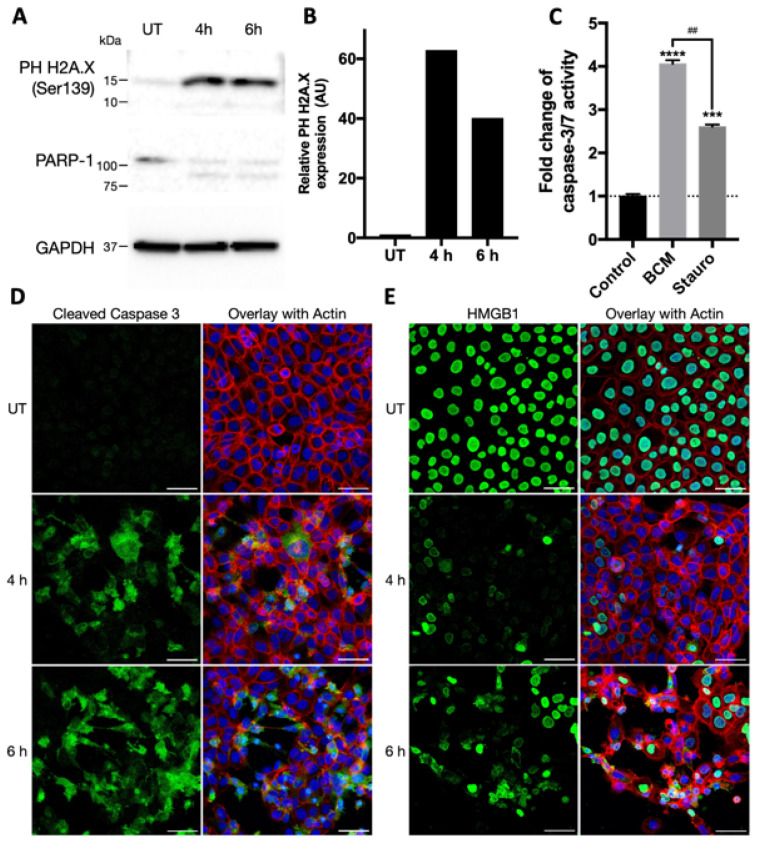
SA29213 BCM reduces metabolic activity and initiates regulated cell death in SCC-12. (**A**) BCM treatment resulted in DNA damage and PARP-1 cleavage in SCC-12. SCC-12 were treated with 2.5 µg/mL BCM. (**B**) Relative protein expression of PH H2A.X of SCC-12 when treated with 2.5 µg/mL BCM. Western blot band intensity was quantified using ImageJ Version 2.14.0/1.54f. Relative expression was normalised to untreated control. (**C**) Caspase 3/7 activity upon treatment with 2.5 µg/mL BCM (BCM) or 0.1 µM staurosporine (Stauro) was assessed via luminescence assay 24 h post-treatment. Luminescence was normalised to untreated control. Data are shown as mean ± SEM of three technical replicates. Two-way ANOVA analyses with post hoc Bonferroni’s multiple comparisons test were performed to control (****: *p* ≤ 0.0001, ***: *p* ≤ 0.001) and between treatments (##: *p* ≤ 0.01) (**D**) Cleaved Caspase-3 is highly expressed in a small population of SCC-12 4 h post BCM treatment. SCC-12 was treated with 2.5 µg/mL BCM. Cell monolayer was labelled for nucleus (blue), cleaved caspase-3 (green) and actin (red). Image acquired using SP8 confocal microscope at 40× magnification. Scale bar represents 50 µm. (**E**) HMGB1 translocates out of the nucleus into the cytoplasm upon BCM treatment. SCC-12 was treated with 2.5 µg/mL BCM. Cell monolayer was labelled for nucleus (blue), HMGB1 (green) and actin (red). Image acquired using SP8 confocal microscope at 40× magnification. Scale bar represents 50 µm.

The involvement of DNA damage, caspase activation, and the regulated cell death pathway in SA29213 BCM-treated SCC-12 was investigated. Treatment with 2.5 µg/mL SA29213 BCM led to the upregulation of the DNA damage marker Phospho-Histone (PH) H2A.X by 4 h post-treatment, indicating the presence of double-stranded DNA breaks in treated SCC-12 (Figure 3A,B). Additionally, the cleavage of Poly (ADP-ribose) polymerase-1 (PARP-1) was observed from 4 h post-treatment, where a band corresponding to the 89 kDa fragment from activated Capases-3 and -7 cleavage becomes apparent. PARP-1 is a nuclear protein involved in the repair of damaged DNA, and its cleavage is frequently involved in the apoptosis signalling cascade [41]. The cleaved catalytic 89 kDa fragment containing an auto-modification domain (AMD) reduced the DNA-binding capacity while the smaller 24 kDa fragment containing a DNA-binding domain (DBD) binds irreversibly with DNA strand breaks, thus inhibiting the actions of active PARP-1 [42]. These suggest that SA29213 BCM induced DNA damage in SCC-12 whilst impairing the DNA repair mechanism through PARP-1 cleavage.

At 24 h post-treatment of SCC-12 with 2.5 µg/mL, SA29213 BCM showed a 4-fold increase in Caspase 3/7 activity. This is higher compared to the approximately 2.6-fold increase in apoptotic SCC-12 induced through 0.1 µM staurosporine treatment (Figure 3C). This corroborates our findings of PARP-1 cleavage in BCM-treated SCC-12. Treatment with 2.5 µg/mL SA29213 BCM led to increased expression in cleaved Caspase-3 (Figure 3D) and the global translocation of HMBG1 out of the nucleus into the cytoplasm by 4 h post-treatment (Figure 3E). This suggests that SA29213 BCM activates the cleaved-Capsase-3-depenedent apoptosis pathway and releases HMGB1 from the nucleus to be released as a damage-associated molecular pattern (DAMP).

**Figure 4:**
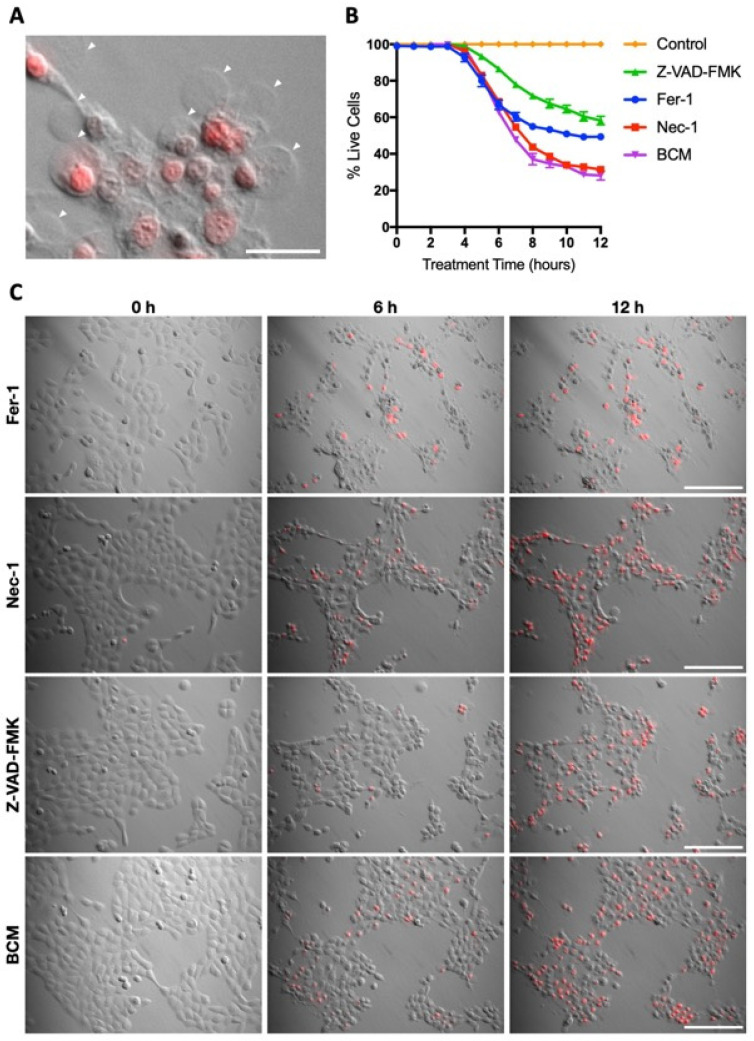
Caspase inhibition delayed SA29213 BCM-induced SCC-12 cell death. (**A**) Morphology of SCC-12 treated with 2.5 µg/mL concentrated SA29213 BCM 18 h post-treatment. White arrow heads highlight giant membrane blebs observed in BCM-induced SCC-12 cell death. Scale bar represents 30 µm. (**B**) Cell death profile of SCC-12 upon BCM treatment was assessed using live/dead fluorescent time-lapse microscopy. Cells were treated with 2.5 µg/mL BCM along with 50 µM of either Fer-1, Nec-1, Z-VAD-FMK, or no inhibitor (BCM). Data are shown as mean ± SEM of three technical replicates. (**C**) Representative images of SCC-12 treated with 2.5 µg/mL BCM along with 100 µM of either Fer-1, Nec-1, Z-VAD-FMK, or no inhibitor (BCM) at 0, 6, 12 h post-treatment. Dead cells were stained with PI (red). Scale bar represents 150 µm.

SA29213 BCM-induced SCC-12 cell death was characterised by the formation of distinct giant membrane blebs (Figure 4A). Unlike membrane disruptions characteristic of apoptosis, SCC-12 cells treated with SA29213 BCM form one or two blebs that could grow up to the original size of the cell. While nuclear condensation was observed in dying SCC-12 cells, nuclear fragmentation characteristic of apoptosis was not observed. The cell death mechanism involved did not appear to strictly fall under Type I cell death. To distinguish the cell death pathway involved in BCM-induced SCC-12 cell death, a panel of cell death inhibitors was employed. Necrostatin-1 (Nec-1), Ferroststain-1 (Fer-1), and carbobenzoxy-valyl-alanyl-aspartyl-[O-methyl]- fluoromethylketone (Z-VAD-FMK) were used to inhibit cell death via necroptosis, ferroptosis, and apoptosis, respectively. Cell death inhibitors were introduced to SCC-12 along with 2.5 µg/mL BCM at the point of treatment. The addition of Z-VAD-FMK at 50 µM caused a delay in BCM-induced SCC-12 cell death, with the percentage of live cells 12 h post-treatment reduced to 60% compared to approximately 30% without inhibitors (Figure 4B). The addition of Nec-1 at 50 µM did not influence the cell death profile of SCC-12 upon BCM treatment. Interestingly, the addition of Fer-1 at 50 µM resulted in the formation of small membrane blebs and a lack of PI staining in blebbing cells, both of which are characteristic of classical apoptosis (Figure 4C). These suggest that the activity of caspase proteases plays a major role in BCM-induced SCC-12 cell death, while features of the ferroptosis pathway may be involved as well. 

### 3.3. Identification of SCC-12-Specific SA29213 Protein Toxin

**Figure 5:**
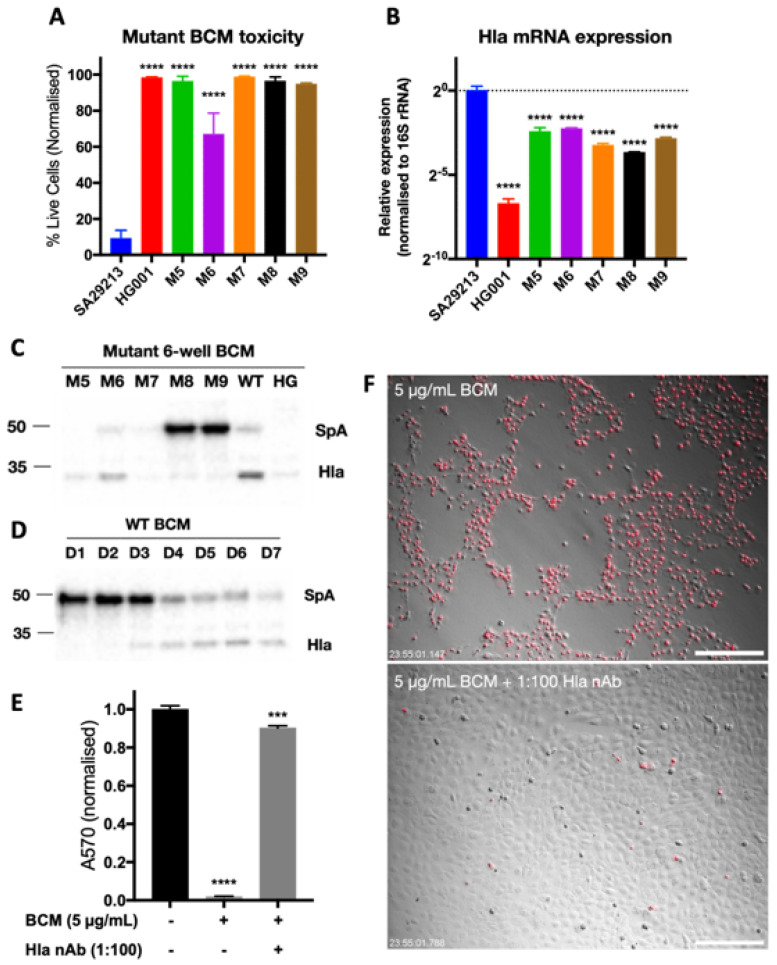
Staphylococcal Hla is the primary source of SCC-12-specific toxicity in SA29213 BCM. (**A**) Effect of SA29213 mutant BCM (M5-8) on SCC-12 cell viability. Cells were treated with 3 μg/mL BCM, and the percentage live cells was assessed by live/dead fluorescence microscopy 24 h post-treatment. (**B**) Hla mRNA fold change of mutant biofilm, normalised to WT SA29213. *n* = 4 (**C**) Western blot of Hla abundance in mutant (M5-8), SA29213 (WT), and HG001 (HG) D3 BCM. BCMs were loaded at equal volumes. (**D**) Western blot of Hla abundance in wild type SA29213 D1-5 BCM. BCMs from various culture days were loaded at equal volumes. (**E**,**F**) Effect of Hla antibody neutralisation on SCC-12 cell viability. Cell were treated with 5 µg/mL BCM with or without 1:100 anti-Staphylococcal Hla rabbit antisera. (**E**) Cell metabolic activity was assessed by MTT assay 24 h post-treatment. (**F**) Representative images of SCC-12 24 h post-treatment taken at 10× magnification. Dead cells were stained with PI (red). Data are shown as mean ± SEM of three technical replicates. Scale bar represents 250 µm. Two-way ANOVA analyses with post hoc Bonferroni’s multiple comparisons test were performed to control (****: *p* ≤ 0.0001, ***: *p* ≤ 0.001).

To identify the SCC-12-specific protein toxin present in SA29213 BCM, a 13,025-membered SA29213 transposon mutant library was constructed. Transposition was conducted using transposon *bursa aurealis* based on the protocol established by Bae et al. [40,43]. Transposon mutants were individually screened for the loss of SCC-12-specific toxicity in BCM generated using a modified high-throughput 96-well protocol. The initial screen identified that 65 mutants had reduced toxicity towards SCC-12, of which 37 were false positives and 10 had reduced growth rates. Of the remaining 18 mutants, 9 produced BCM with low protein concentrations. BCM was generated for the remaining nine mutants using a modified 6-well protocol, and the toxicity of the mutant BCMs towards SCC-12 was verified. D3 BCM of all mutants except mutants 4 and 6 showed negligible toxicity towards SCC-12 24 h post-treatment when applied at 3 µg/mL (Figure 5A). The *S. aureus* strain HG001, which produces BCM with low and no toxicity towards SCC-12, was included as controls.

LC-MS/MS of wild-type (WT) SA29213 BCM ion-exchange fast protein liquid chromatography (FPLC) fractionation identified several proteins as present in the fraction retaining toxicity towards SCC-12. The mRNA expression levels of the corresponding genes in each mutant were quantified through RT-qPCR using RNA isolated from D3 bacteria biofilms. Of the genes of interest studied, the expression of alpha hemolysin (Hla) was significantly reduced in most of the identified mutants (Figure 5B). Western blotting of mutant BCM also showed a reduction in Hla in mutants generating BCM with low toxicity towards SCC-12 (Figure 5C). In addition, the protein expression of Hla increases with prolonged culturing in WT SA29213, with expression notably high in BCM after 4 days of culturing (Figure 5D). The addition of anti-staphylococcal Hla attenuates BCM-induced cellular toxicity, with a rescue of metabolic activity to 90.21% of untreated control (Figure 5E) and near-complete eradication of cell death (Figure 5F).

### 3.4. Characterisation of Hla as an SCC-12-Specific Protein Toxin

**Figure 6:**
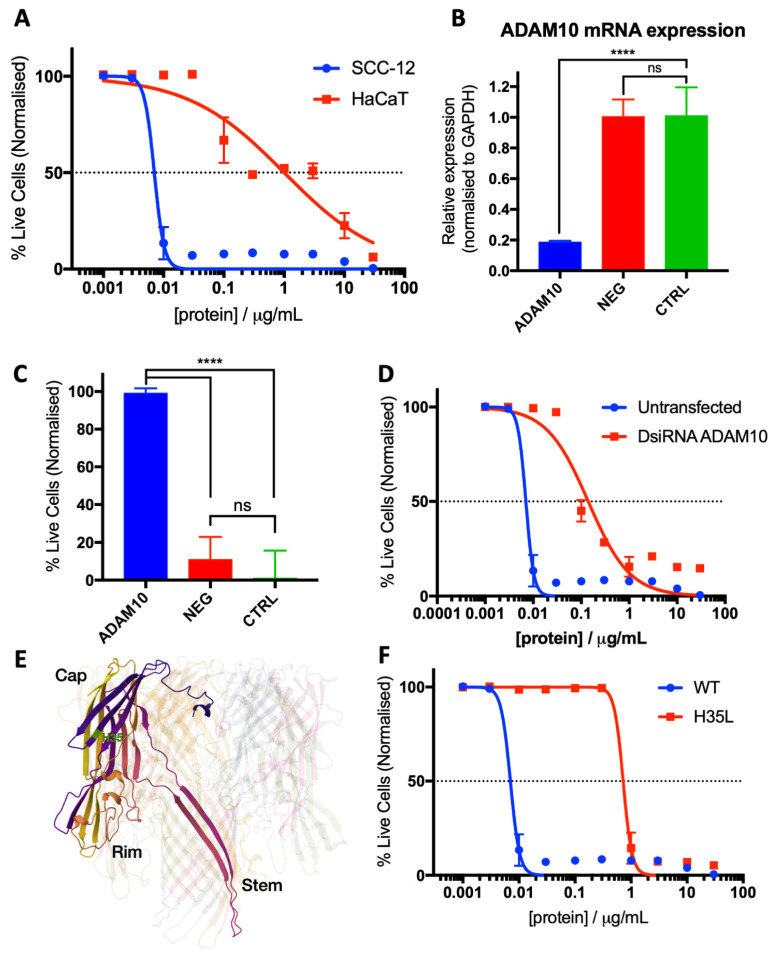
Staphylococcal Hla is the primary source of SCC-12-specific toxicity in SA29213 BCM. (**A**) Cell death profile of SCC-12 and HaCaT upon Hla treatment was assessed using live/dead fluorescent time-lapse microscopy. Cells were treated with 0.001 to 30 µg/mL purified Hla. Line represents non-linear regression. *n* = 3. (**B**) ADAM10 mRNA expression in DsiRNA ADAM10 trans-fected cells (ADAM10), sham transfected (NEG) and untransfected cells (CTRL) 48 h post-transfection. (**C**,**D**) ADAM10 knockdown increases SCC-12 tolerance towards Hla. (**C**) Percentage live cells of DsiRNA ADAM10 transfected cells (ADAM10), sham transfected (NEG) and untransfected cells (CTRL) treated with 2.5 µg/mL SA29213 BCM was assessed by live/dead fluorescence microscopy 24 h post-treatment. Ordinary one-way ANOVA analyses with post hoc Dunnett’s multiple comparisons test of conditions were performed with respect to ADAM10 transfected cells (****: *p* < 0.0001; ns: *p* > 0.05). (**D**) Cell death profile of DsiRNA ADAM10 transfected and untransfected SCC-12 upon Hla treatment was assessed using live/dead fluorescent time-lapse microscopy. Cells were treated with 0.001 to 30 µg/mL purified Hla. Line represents non-linear regression. (**E**) Ribbon diagram of Hla homoheptamer, with a single chain highlighted. Cap, stem, and rim domains of Hla monomer are annotated. The amino acid residue H35 is highlighted in green. Image adapted from Song et al., 1996 [44] (PDB 7AHL). (**F**) Cell death profile of SCC-12 upon WT or H35L Hla treatment was assessed using live/dead fluorescent time-lapse microscopy. Cells were treated with 0.001 to 30 µg/mL purified Hla. Line represents non-linear regression. *n* = 3.

The effect of Hla in isolation on the cell viability of cancerous keratinocyte (SCC-12) and non-cancerous keratinocyte (HaCaT) cell lines was assessed. Purified SA29213 Hla was produced in collaboration with the NTU Protein Purification Platform (NTU-PPP). The CC50 of each cell line was determined by quantitating the degree of cell death upon treatment with purified Hla for 24 h. SCC-12 displayed a much higher susceptibility (CC50 = 7.031 ng/mL) towards Hla than non-cancerous HaCaT (CC50 = 1004 ng/mL) (Figure 6A). The selectivity index (SI) of Hla on SCC-12 was 142.8.

To understand the mechanism behind Hla toxicity in SCC-12, the role of Hla-cell surface ligand interaction was investigated. In human tissues, Hla specifically binds to cell the surface ligands disintegrin and metalloprotease 10 (ADAM10) at low concentrations [45]. The essential nature of this interaction in Hla-induced SCC-12 cell death was investigated though short-interfering RNA (siRNA) knockdown of ADAM10.

Reverse transfection of 10 nM ADAM10 Dicer-substrate siRNAs (DsiRNAs) resulted in an approximately 80% reduction in ADAM10 mRNA expression 48 h post-transfection (Figure 6B). This is sufficient to attenuate SCC-12 susceptibility towards 2.5 µg/mL SA29213 BCM challenge, a concentration lethal towards untransfected SCC-12 (Figure 6C). Similarly, ADAM10 knockdown resulted in an approximately 20-fold increase in tolerance towards purified Hla in SCC-12 (Figure 6D). This suggests that Hla-ADAM10 interaction, while not essential, allows Hla to exert its toxicity with ADAM10-dependent cell-type specificity.

The essential nature of Hla oligomerisation and transmembrane pore formation was also explored. Hla with a single amino acid modification H35L, a mutation, which results in reduced pore-forming and oligomerisation abilities [27,46,47,48,49,50,51,52], was used in this study (Figure 6E). A purified SA29213 Hla H35L oligomerisation-deficient mutant was produced in collaboration with the NTU Protein Purification Platform (NTU-PPP).

The loss of oligomerisation and pore-forming abilities drastically reduced Hla toxicity towards SCC-12. H35L is approximately 100-times less toxic than WT Hla towards SCC-12, as assessed through live/dead florescence microscopy (Figure 6F). This strongly suggests that Hla oligomerisation and pore formation are important for efficient death induction in SCC-12.

## 4. Discussion

In this study, we described a highly efficient and cost-effective way of generating *S. aureus* BCM for the study of *S. aureus* biofilm-associated soluble products. The protocol allows for good accumulation of the biofilm that is easily observable and quantifiable. The use of easily attainable aquaria filter wool as a scaffold for the enrichment of biofilms makes the protocol much more accessible compared to the use of trans-well inserts [53,54,55,56,57] and flow reactor systems [58] in previously published protocols. In addition, the culturing of biofilms on aquaria wool, which could be easily sampled, enables simple and reproducible quantification of the *S. aureus* biofilm compared to other biofilm quantification assays such as the crystal violet microtiter dish biofilm formation assay. Such assays involve fixation, staining, and extensive washing, which renders them difficult to reproduce due to the fragility for *S. aureus* biofilms. This method is useful in the easily scalable generation of biofilm-conditioned media and as an indicator of biofilm-forming ability.

*S. aureus* clinical strain SA29213 BCM possess high specific toxicity towards SCC, which is conferred by *S. aureus* alpha hemolysin (Hla). SA29213 BCM and Hla are more than 20- and 140-times more toxic towards the SCC cell line SCC-12 than the noncancerous keratinocyte cell line HaCaT, respectively. The BCM-induced SCC-12 cell death also did not cause secondary toxicity towards HaCaT, suggesting the potential of Hla to have low off-target effects when used in isolation in vivo. To date, this is the first report of an *S. aureus* toxin as a potential skin cancer therapeutic with cancer-specific cytotoxicity towards SCC. *S. aureus* BCM were previously described to hamper keratinocyte migration, suggesting their involvement in a chronic wound’s inability to heal [59]. We did see that SA29213 BCM reduce the metabolic activity of HaCaT at non-lethal doses. The extent of the reduction, however, does not inhibit HaCaT proliferation. In SCC-12/HaCaT co-culture, HaCaT was observed to be proliferative when treated with SA29213 BCM at a dose lethal to SCC-12. The *S. aureus* secretome has been well documented, with the mechanism of action of most of the virulence factors established. However, none have been described to cause cell-line-specific toxicity to date. 

BCM-treated SCC-12 also undergoes a cell death pathway that is unlike classical apoptosis. Our data show that treatment with BCM caused DNA double-stranded breaks, a global migration of HMGB1 into the cytoplasm, activation of Caspase 3/7, and expression of cleaved capase-3 in SCC-12 cells. There is also a marked difference in morphology in the cell death process in BCM-treated SCC compared to cells dying via classical apoptosis, with giant membrane blebs forming at the cell periphery and PI uptake. This distinct morphology points towards regulated cell death pathway ferroptosis and pyroptosis to be the potential modes of cell death, both of which induce the production of large membrane blebs in dying cells. However, the ferroptosis inhibitor Ferrostatin-1 failed to delay cell death in SA29213 BCM-treated SCC-12. In addition, we did not observe the Caspase 1 and inflammasome activation that is distinct in pyroptotic cells, which has been previously observed in Hla-treated cells [60,61]. The elucidation of the SA29213 BCM-induced cell death mechanism requires further investigation.

Our findings demonstrate that *S. aureus* alpha hemolysin (Hla) is the primary agent within SA29213 BCM causing SCC-12-specific cell death. The protein expression of Hla corresponds to the extent of toxicity towards SCC-12, and the addition of anti-Hla whole sera effectively neutralises SA29213 BCM toxicity. The use of Hla in cancer therapeutics has been previously explored, albeit only through live bacterial delivery. *S. aureus* Hla delivered using a live *E. coli* vector was demonstrated to induce carcinoma cell death and reduce tumour volume in murine mammary carcinoma [62,63]. Hla has also been shown to confer toxicity towards cisplatin-resistant malignant pleural mesothelioma cells and re-sensitize the cells towards cisplatin [64]. However, the cancer cell specificity of native Hla has not been previously reported. This study demonstrates the innate cancer-cell-associated target specificity of Hla, as seen from the high target specificity of SA29213 BCM. Although the use of Hla as a cancer therapeutic comes with significant risk, demonstrations of targeted Hla delivery to tumours in vivo [62,65] suggest that Hla, when delivered in a controlled manner, could be effective in cancer killing while exerting minimal systemic effects. 

To explore Hla’s application as a targeted cancer therapeutic, the mechanism linking Hla pore formation, oligomerisation, ADAM10 binding, and the eventual caspase-dependent cell death needs to be elucidated. The question of whether cellular injury resulting from pore formation and ADAM10 pathway activation is essential in cell death initiation needs to be addressed. This would potentially open doors to generating mutant Hla that initiates a target-specific regulated cell death pathway with low off-target toxicities. The structure of Hla, and the role of specific domains, has been well studied [44,66,67,68,69,70,71], and a combination of protein truncation and domain modification could produce a mutant Hla bearing just the essential properties for the initiation of targeted cell death.

Hla binds specifically to the human cell surface ligands disintegrin and metalloprotease 10 (ADAM10) at low concentrations [45]. ADAM10 is ubiquitously expressed in humans [72] and influences over 40 substrates, of which a portion is involved in cancer proliferation [73]. Elevated levels of ADAM10 have been associated with poor prognosis in cancers such as glioblastoma and breast cancer [74,75,76], and ADAM10 shows potential as a pan-cancer biomarker [77]. Interestingly, ADAM10 upregulation appears to play an important role in disease progression in various squamous cell cancers, such as oral SCC [78,79], tongue SCC [80], and non-small cell lung cancer [81]. The regulation of ADAM10 in cutaneous SCC is not well understood; however, Oh et al. published a letter in 2016 providing histological evidence of ADAM10 and ADAM17 upregulation in SCC [82]. Physiological ADAM10 expression protects against skin infection [83]. ADAM10 targeting has also been explored in cancers, such as glioblastoma, Hodgkin lymphoma, non-Hodgkin lymphoma, multiple myeloma, and breast cancer (reviewed in [74,84]). Several compounds modulating the expression and activity of ADAM10 have been investigated for their therapeutic potential in clinical trials [84], including the use of Rapamycin for the inhibition of ADAM10 activity in Alzheimer disease [85]. However, the use of ADAM10 for cancer cell targeting has not been undertaken yet. Our study suggests that ADAM10 is the molecular basis of SA29213 BCM’s specific toxicity and that ADAM10 could potentially be used as an SCC therapeutic target.

## 5. Conclusions

The relationship between human skin and *S. aureus* is extremely complex, and significant work is required to elucidate the intricate web of host–bacteria crosstalk that occurs in both healthy and diseased skin. Here, we show a counterintuitive effect of *S. aureus* biofilm-associated secreted factors on cancerous keratinocytes. The highly target-specific nature and the potential of having low off-target effects make Hla a worthy SCC therapeutic candidate. Future investigations of Hla would support the ongoing efforts in the pursuit of a non-surgical alternative in SCC therapy. 

## Data Availability

The data presented in this study are available on request from the corresponding author.
